# Runx2 overexpression promotes bone repair of osteonecrosis of the femoral head (ONFH)

**DOI:** 10.1007/s11033-023-08411-7

**Published:** 2023-04-07

**Authors:** Hai-Jia Xu, Xiang-Zhong Liu, Lu Yang, Yu Ning, Liang-Liang Xu, Da-Ming Sun, Wen Liao, Yi Yang, Zhang-Hua Li

**Affiliations:** 1 Department of Orthopedics, Wuhan Third Hospital, Tongren Hospital of Wuhan University, Wuhan, 430060 China; 2 Department of Anesthesiology, Wuhan Third Hospital, Tongren Hospital of Wuhan University, Wuhan, 430060 China; 3 Department of Orthopedics, XiangYang Hospital of Traditional Chinese Medicine, Hubei University of Chinese Medicine, Xiangyang, 441000 China; 4 Wuhan Sports University, Wuhan, 430079 China

**Keywords:** Osteonecrosis of the femoral head, Mesenchymal stem cells, Runx2, Bone repair

## Abstract

**Background:**

Runt-related transcription factor-2 (Runx2) has been considered an inducer to improve bone repair ability of mesenchymal stem cells (MSCs).

**Methods and results:**

Twenty-four rabbits were used to establish Osteonecrosis of the femoral head (ONFH) and randomly devided into four groups: Adenovirus Runx2 (Ad-Runx2) group, Runx2-siRNA group, MSCs group and Model group. At 1 week after model establishment, the Ad-Runx2 group was treated with 5 × 107 MSCs transfected through Ad-Runx2, the Runx2-siRNA group was treated with 5 × 107 MSCs transfected through Runx2-siRNA, the MSCs group was injected with 5 × 107 untreated MSCs, and the Model group was treated with saline. The injection was administered at 1 week and 3 weeks after model establishment. The expression of bone morphogenetic protein 2 (BMP-2), Runx2 and Osterix from the femoral head was detected at 3 and 6 weeks after MSCs being injected, and Masson Trichrome Staining, Gross Morphology, X-ray and CT images observation were used to evaluate the repair effect of ONFH. The data revealed that the expression of BMP-2, Runx2 and Osterix in the Runx2-siRNA group was reduced at 3 weeks compared with the MSCs group, and then the expression further reduced at 6 weeks, but was still higher than the Model group besides Osterix; The expression of these three genes in the Ad-Runx2 group was higher than in the MSCs group. Masson Trichrome Staining, Gross Morphology and X-ray and CT images observation revealed that necrotic femoral head of the MSCs group was more regular and smooth than the Runx2-siRNA group, which has a collapsed and irregular femoral head. In the Ad-Runx2 group, necrotic femoral head was basically completely repaired and covered by rich cartilage and bone tissue.

**Conclusions:**

Overexpression of Runx2 can improve osteoblastic phenotype maintenance of MSCs and promote necrotic bone repair of ONFH.

## Introduction

ONFH is a common bone disease featured by interruption of angiogenesis, bone cell and bone marrow death, bone collapse, with about 75% of patients aged 30 to 60 years. Its etiology is very complex, including but not limited to trauma, overdose of glucocorticoids administration and excessive drinking [1–4]. Early diagnosis and treatment can prevent the progression of joint destruction, reduce the implementation of traumatic surgery, and improve the probability of hip preservation, such as some lncRNA, ultrasound molecular imaging, core decompression, osteotomy and physical therapy, etc. However, these methods are usually not very satisfactory, approximately 40% of patients progressing to total hip arthroplasty [5–8]. MSCs are considered to be an ideal material for bone regeneration and vascular regeneration because of its differentiation potency [9, 10].

In recent years, stem cells transplantation has been one of the most common methods for the treatment of ischemic diseases [11]. Mastrolia and and his colleagues confirmed bone formation through transplanting autologous MSCs to treat ONFH [12]. In our previous experiments, a large number of high-purity allogeneic MSCs have been confirmed to migrate, survive and proliferate in necrotic bone, and will not cause acute or chronic immune rejection and manifestations of graft-versus-host disease [13]. Further animal experiments confirmed that allogeneic MSCs can improve osteonecrosis blood supply and promote bone regeneration [14]. However, Simple MSCs transplantation have some shortcomings, such as a large number of cells are needed, multiple transplants are needed, long time to repair is needed and unstable differentiation ability without induction. Some methods have been used to induce MSCs differentiate into osteoblasts, These methods mainly include drugs, physical stimulation, exosomes, gene transfection and platelet lysate and so on [15–22].

As the core binding factor a1 (Cbfa1), Runx2 belongs to the RUNT transcription factor family which is expressed in MSCs and osteochondral progenitor cells, performing as a “master” regulator of osteogenesis [23–25]. But there is not direct evidence that MSCs overexpressing Runx2 can repair ONFH. In this paper, MSCs were injected into ONFH rabbit model after being transfected with Adenovirus Runx2, and histological, radiologic and molecular analyses were used to detect the repair effect.

## Materials and methods

### MSCs isolation and identification

MSCs were isolated according to the previous method [26]. In brief, bone marrow of the four limbs was obtained by flushing with 26-gauge syringe needle containing phosphate-buffered saline (PBS) under sterile conditions. The contents were washed and centrifuged at 1500 rpm for 5 min. And then MSCs was resuspended with mesenchymal stem cell medium (MSCM, Sciencell. USA) and maintained in a 37 ºC incubator with 5% CO2. MSCs were observed under a fluorescence inverted microscope (Leica. Germany) and passaged when they reached 80–90% confluence.

For phenotypic characterization, 5 × 10^5^ MSCs were trypsinized and washed twice with PBS, and were incubated with fluorescein isothiocyanate (FITC)-conjugated anti-CD29 (ab255354), anti-CD44 (ab189524) and anti-CD45 (ab40763) (all from Abcam, USA) at a dilution rate of 1: 500 for 30 min at 4 °C. Then, MSCs were washed twice with PBS again and analyzed by CytoFLEX S Flow cytometry (Beckman, USA).

For adipogenic and osteogenic differentiation, 2 × 10^4^ MSCs were seeded in a 6-well plate and maintained with osteogenic differentiation medium and adipogenic differentiation medium (Cyagen, USA) for 3 weeks. Mineralized nodule and lipid droplets were detected by alizarin red and Oil red O staining kit (Cyagen, USA), and observed under a fluorescence inverted microscope.

### Ad-Runx2 and Runx2-siRNA transfection

Third passage MSCs were transfected with Ad-Runx2 (was synthesized in Shanghai Jikai Biotech Co., Ltd) and Runx2-siRNA (was synthesized in Guangzhou Ribobio Biotech Co., Ltd) according to manufacturer’s instructions and previous research [26]. Briefly, Ad-Runx2 with Green Fluorescence Protein (GFP) fluorescence was mixed with Dulbecco’s Modified Eagle’s Medium (DMEM, meilunbio, China) and was added to MSCs followed by incubation for 6 h. Then, MSCM was added followed by incubation for 72 h. Runx2-siRNA with CY3 fluorescence and lipofectamine 2000 (Thermo fisher. USA) were diluted by DMEM for 5 min, and they are mixed followed by incubation for 20 min. Then, the mixture was added to MSCs followed by incubation for 72 h.

### Western blot analysis

Western blot analysis was used to detected the protein level of BMP-2 (ab6285), Runx2 (ab114133), Osterix (ab94744) (all from Abcam, USA) and glyceraldehyde-3-phosphate dehydrogenase (GAPDH, ab9485). Total protein was extracted and its concentration was measured by BCA assay kit (Servicebio, China) followed by mixing with 5×loading buffer at 95 °C for 5 min. Total protein of each group was separated by 10% SDS-PAGE at 60 V for 30 min and 100 V for 1 h, and transferred to a PVDF membrane (Millipore, USA) at 200 mA for 1 h. The PVDF memberane was blocked with 5% skim milk for 1 h and incubated with primary antibodies against BMP-2 (1:2000), Runx2 (1:1000), Osterix (1:2000) and GAPDH (1:5000) at 4 °C overnight. The memberane was incubated with secondary antibodies (1:7000) at room temperature for 1 h after being washed three times with washing buffer. Then, The member was incubated with enhanced chemiluminescence (Biorad, USA) and exposed by the gel imaging system (Biorad, USA). GAPDH was employed as the internal reference and each experiment was performed in triplicate. The Image J software was used for grayscale determination.

### Quantitative real-time PCR (qPCR) analysis

Total RNA was extracted by the Trizol RNA Extracting Solution (TOYOBO, Japan) and reverse-transcribed into cDNA by a cDNA synthesis kit (TOYOBO, Japan). The relative mRNA expression levels of BMP-2, Runx2, Osterix and GAPDH were detected through SYBR Green Realtime PCR Master mix (TOYOBO, Japan). The primer sequences were synthesised at SANGON Biotech Co, Ltd (Shanghai, China). The primer sequences are shown in Table 1.

qPCR was performed by the Bio-Rad CFX Manager system (Bio-Rad, USA), including 10 µL of 2 × uitraSYBR mixture, 1 µL of forward primer and reverse primer, 1 µL of cDNA and ddH2O 7 µL. Reaction conditions were as follows: pre-denaturation at 95 °C for 3 min, 40 cycles of denaturation at 95 °C for 10 s, annealing at 60 °C for 10 s and extending at 72 °C for 10 s. The relative expression of mRNAs was calculated by the 2^−∆∆Ct^. GAPDH was employed as the internal reference and each experiment was performed in triplicate.

**Table 1:** Primer sequences

Forward Primer (5′-3′)	Reverse Primer (5′-3′)
BMP-2 TGTGGACTTCAGTGATGTG	TGGAGTTCAGGTGGTCAG
Runx2 GACTGTGGTTACCGTCATGGC	ACTTGGTTTTTCATA ACAGCGGA
Osterix TCCCTGGATATGACTCATCCCT	CCAAGGAGTAGGTGTGTTGCC
GAPDH CTGGGCTACACTGAGCACC	AAGTGGTCGTTGAGGGCAATG

### Model establishment and MSCs transplantation

Twenty-four rabbits (body weight 2.5 ± 0.5 kg) provided by the Animal Facility ofthe Wuhan wan qian jia xing Biotech Co., Ltd (Lenience No. SCXK Hubei 2019-0011), without a specific requirement for sex. They were raised in a single cage with a normal diet. These rabbits were randomly devided into four groups: Ad-Runx2 group, Runx2-siRNA group, MSCs group and Model group.

The rabbit model of femoral head necrosis was established according to the previous method [18]. Briefly, the articular surface of femoral head was completely exposed under sterile conditions and treated with liquid nitrogen for 3–5 min until the color of the articular cartilage turned pale. Femoral head was immediately re-warmed in saline at 37 °C for 3 min. The wound was closed and sutured. Then 800 KU of penicillin was injected intramuscularly for 5 consecutived days.

At 1 week after model establishment, 5 × 10^7^ MSCs transfected with Ad-Runx2 (about 0.4 mL of cell solution), 5 × 10^7^ MSCs transfected with Runx2-siRNA (about 0.4 mL of cell solution), 5 × 10^7^ MSCs (about 0.4 mL of cell solution) and 0.4 mL saline were intravenously injected into Ad-Runx2 group, Runx2-siRNA group, MSCs group and Model group respectively. The injection was administered at the 1 week and 3 weeks after model establishment. The femoral head was detected at the 3 and 6 weeks after MSCs being injected.

### X-ray and Micro CT Imaging

X-ray and Micro CT were taken to detect the necrotic femoral head. The scanning parameters of Micro CT were 5 mm thickness, 120 kV tube voltage and 60 mA tube current. Imaging analysis was used for qualitative observation of lesion location, size, conformation, marginal and internal structure, and secondary changes.

### Statistical analysis

Data were expressed as mean ± standard deviation (SD) and analyzed with SPSS 23.0 statistical software (SPSS, Chicago, IL, USA). Comparisons among groups was conducted with one-way ANOVA. *P* < 0.05 was considered a statistically significant difference.

## Results

### Identification of MSCs

**Fig. 1:**
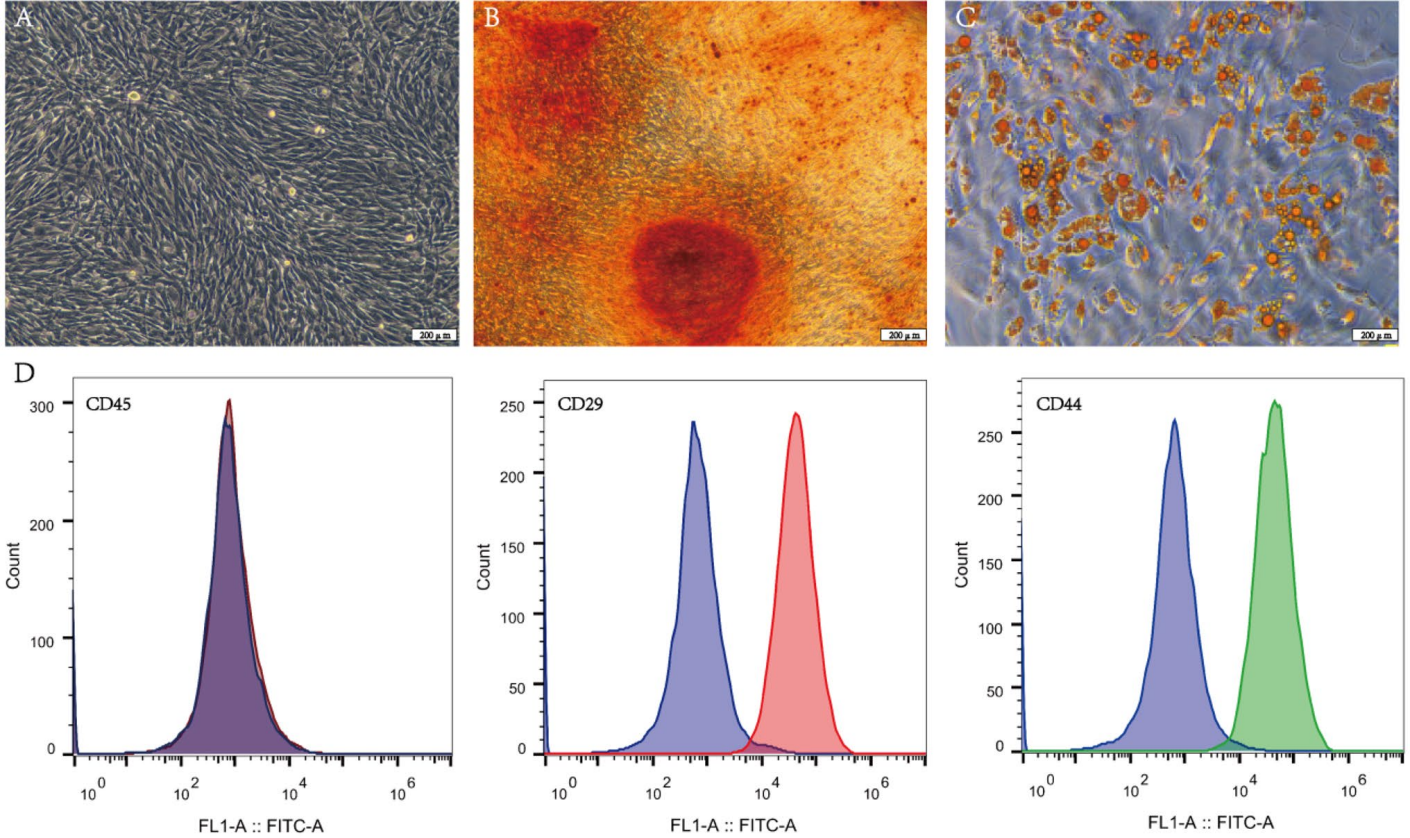
Cultivation and identification of MSCs. ( The morphology of P3 MSCs was observed by microscopy. **B** The mineralized nodules of MSCs were screened by alizarin red staining. **C** The lipid droplets of MSCs were screened by Oil red O staining. **D** Flow cytometry was used to identify cell surface markers (CD29, CD44, CD45)

MSCs were exhibited whirl-like growth under low-power microscope, and displayed long spindle-shaped and fibroblast-like under high-power microscope (Fig. 1A). After adipogenesis and osteogenesis induction for 3 weeks, Oil red O staining showed lipid droplets, and alizarin red staining showed mineralized nodule (Fig. 1B, C). Flow cytometry showed that MSCs were positive for CD29 and CD44, but negative for CD45 (Fig. 1D).

### Transfection efficiency test of Ad-Runx2 and Runx2-siRNA

**Fig. 2:**
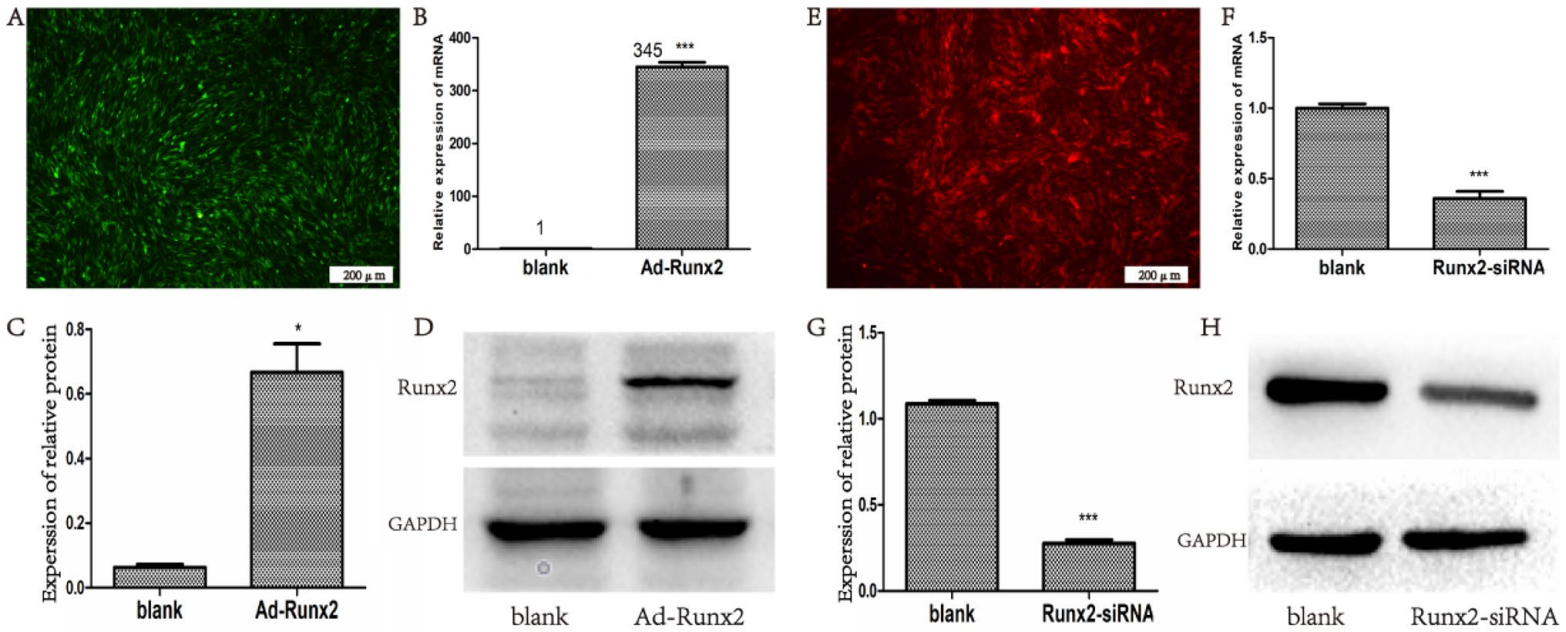
Ad-Runx2 and Runx2-siRNA transfection efficiency test. **A** and **E** The GFP and CY3 fluorescent distribution of MSCs was observed. **B** and **F** The mRNA level of Runx2 determined by qPCR. **C**, **D**, **G** and **H** The protein level of Runx2 measured by Western blot analysis. *P < 0.05 and ***P < 0.001 compared with the blank group respectively

Green and red fluorescence from GFP and CY3 were observed evidently under a fluorescence inverted microscope (Fig. 2A and E). qPCR and Western blot were used to detect the mRNA and protein levels of Runx2 respectively. These results demonstrated that compared with the blank group, the Ad-Runx2 transfection group exhibited a significantly increased expression of Runx2 (*P* < 0.001) (Fig. 2B, D), the Runx2-siRNA transfection group exhibited a completely inhibited effect (*P* < 0.001) (Fig. 2F, G, H).

### Runx2 affected the osteogenic differentiation of MSCs

**Fig. 3:**
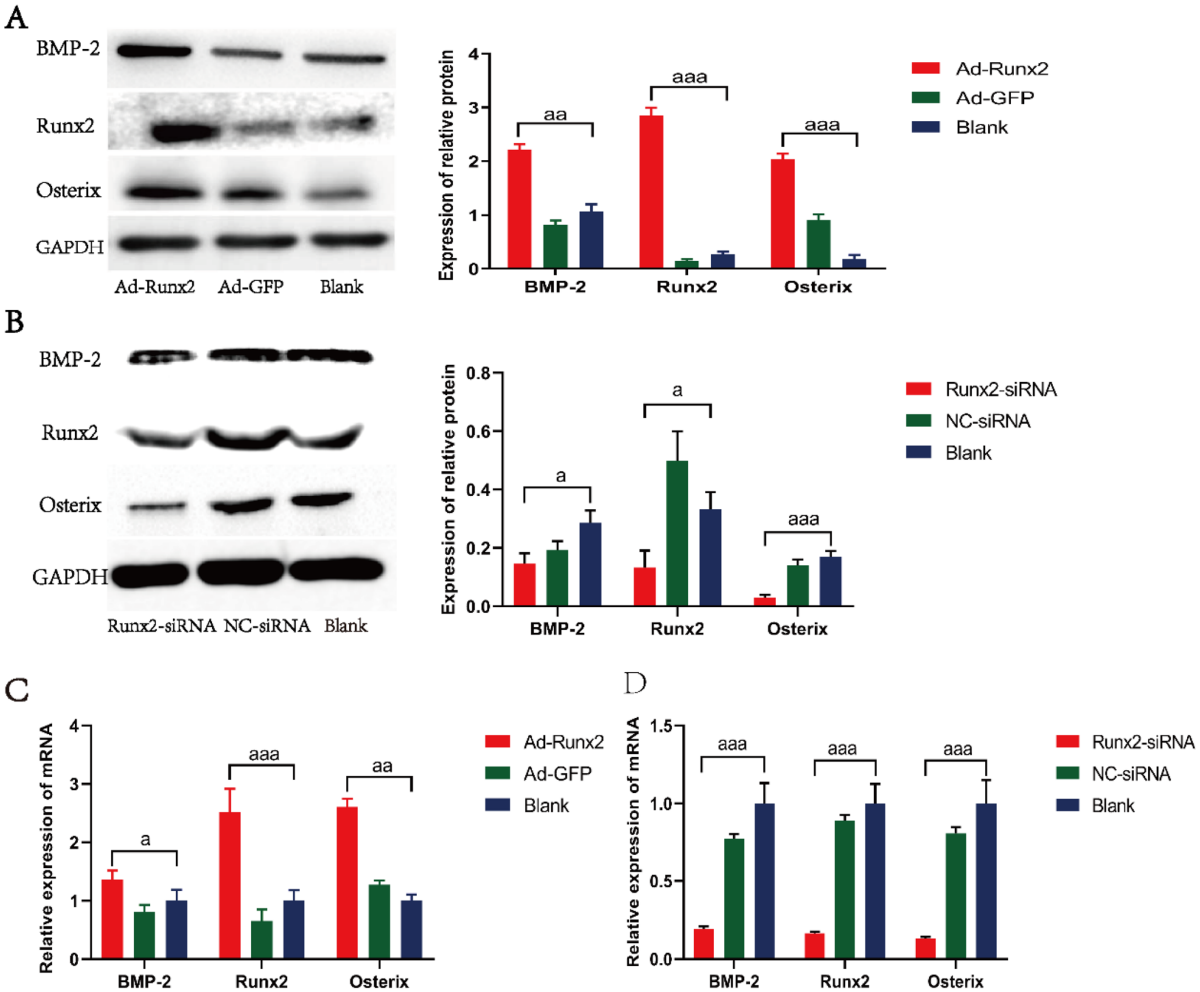
Runx2 overexpression induces osteogenic differentiation of MSCs. **A** and **B** The BMP-2, Runx2 and Osterix determined by Western blot; **C** and **D** The BMP-2, Runx2 and Osterix determined by qPCR; ^a^*P* < 0.05, ^aa^*P* < 0.01 and ^aaa^*P* < 0.001 compared with the blank group;

Western blot and qPCR were used to detect the expression of osteoblastic phenotype after MSCs being transfected by Ad-Runx2 and Runx2-siRNA. The results of Western blot and qPCR demonstrated that compared with the blank group, the Ad-Runx2 transfection group exhibited a significantly increased expression of BMP-2, Runx2 and Osterix (*P* < 0.05) (Fig. 3A C), the Runx2-siRNA transfection group exhibited a significantly decreased expression of BMP-2, Runx2 and Osterix (*P* < 0.05) (Fig. 3B and D). These results showed that Runx2 overexpression can induce the osteogenic differentiation of MSCs.

### Overexpression of Runx2 promotes the expression of osteoblastic phenotype

**Fig. 4:**
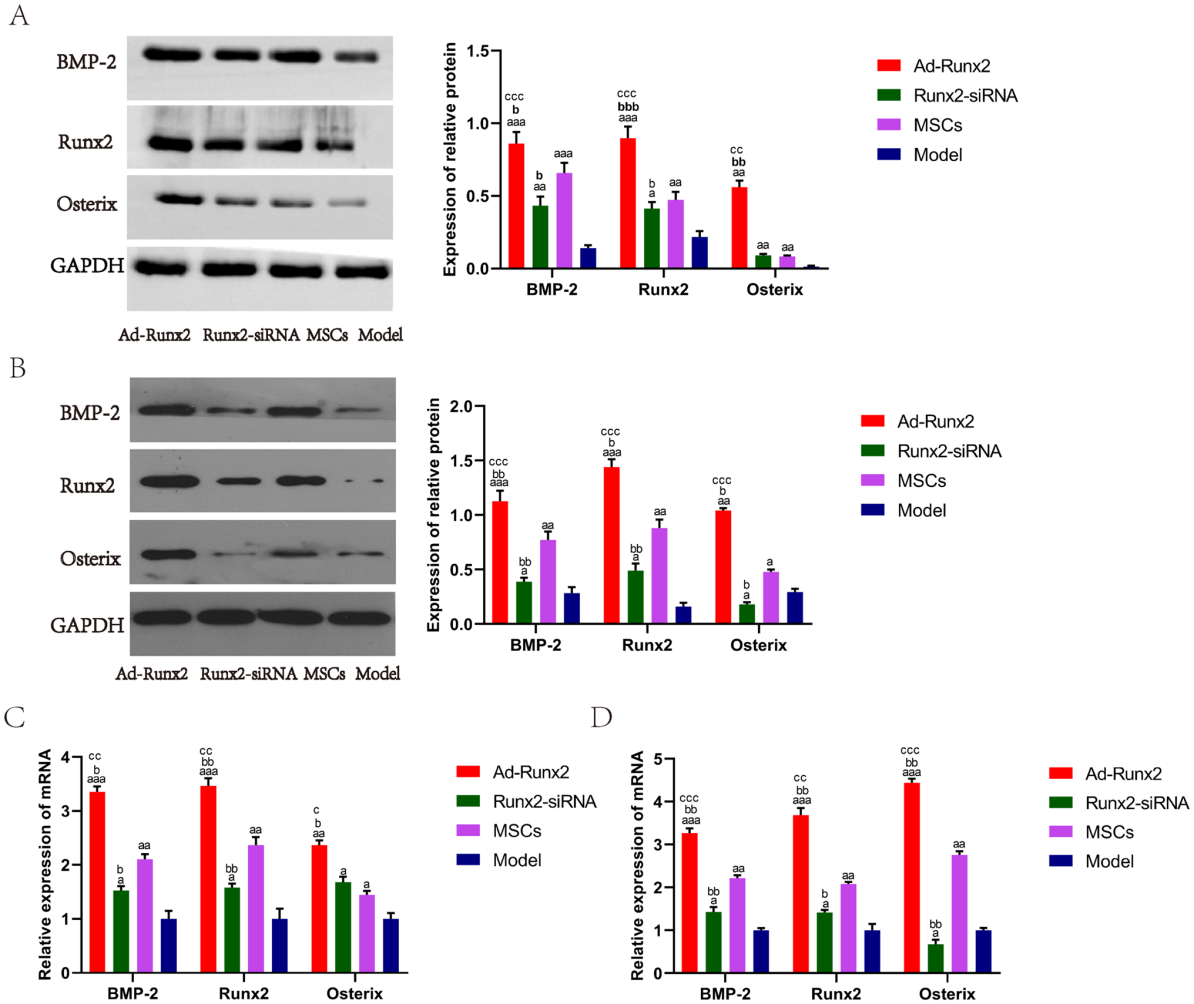
Up-regulated Runx2 accelerates osteonecrosis repair at 3 weeks and 6 weeks. **A** and **B** The BMP-2, Runx2 and Osterix determined by Western blot; **C** and **D** The BMP-2, Runx2 and Osterix determined by qPCR; ^a^*P* < 0.05, ^aa^*P* < 0.01 and ^aaa^*P* < 0.001 compared with the Model group; ^b^*P* < 0.05, ^bb^*P* < 0.01, ^bbb^*P* < 0.001 compared with the MSCs group; ^c^*P* < 0.05, ^cc^*P* < 0.01, ^ccc^*P* < 0.001 compared with the Runx2-siRNA group

qPCR and Western blot were used to detect the expression of osteoblastic phenotype from the femoral head at 3 and 6 weeks after MSCs being injected into ANFH models. The results of Western blot at 3 weeks revealed that compared with the Model group, the expression of BMP-2, Runx2 and Osterix were up-regulated significantly in the Ad-Runx2, Runx2-siRNA and MSCs groups (*P* < 0.05). Compared with the MSCs group, the Ad-Runx2 group presented a significantly augmented BMP-2, Runx2 and Osterix (*P* < 0.05); However, the Runx2-siRNA group showed a significant inhibition of BMP-2 and Runx2 (*P* < 0.05), and no difference of Osterix (Fig. 4A ). The results of Western blot at 6 weeks revealed that compared with the Model group, the expression of BMP-2 and Runx2 were up-regulated significantly, and the expression of Osterix was down-regulated in the Runx2-siRNA group (*P* < 0.05). Compared with the MSCs group, the Ad-Runx2 group presented a significantly augmented BMP-2, Runx2 and Osterix (*P* < 0.05); However, the Runx2-siRNA group showed a significant inhibition of BMP-2, Runx2 and Osterix (*P* < 0.05) (Fig. 4B ). These results suggested that Runx2 overexpression can promote osteoblastic phenotype maintenance.

qPCR analysis at 3 weeks showed that compared with the Model group, the Ad-Runx2, Runx2-siRNA and MSCs groups exhibited significantly increased level of BMP-2, Runx2 and Osterix. Compared with the MSCs group, the expression of BMP-2, Runx2 and Osterix were further increased in the Ad-Runx2 group (*P* < 0.05); However, the Runx2-siRNA group showed a significant inhibition of BMP-2 and Runx2 (*P* < 0.05), and no difference of Osterix (Fig. 4C ). qPCR analysis at 6 weeks showed compared with the Model group, the Runx2-siRNA group presented a significantly augmented BMP-2 and Runx2, and a significantly decreased Osterix (Fig. 4D ). The results of qPCR analysis were basically consistent with Western blot.

### Masson trichrome staining, gross morphology and images observation findings of osteonecrosis repair

**Fig. 5:**
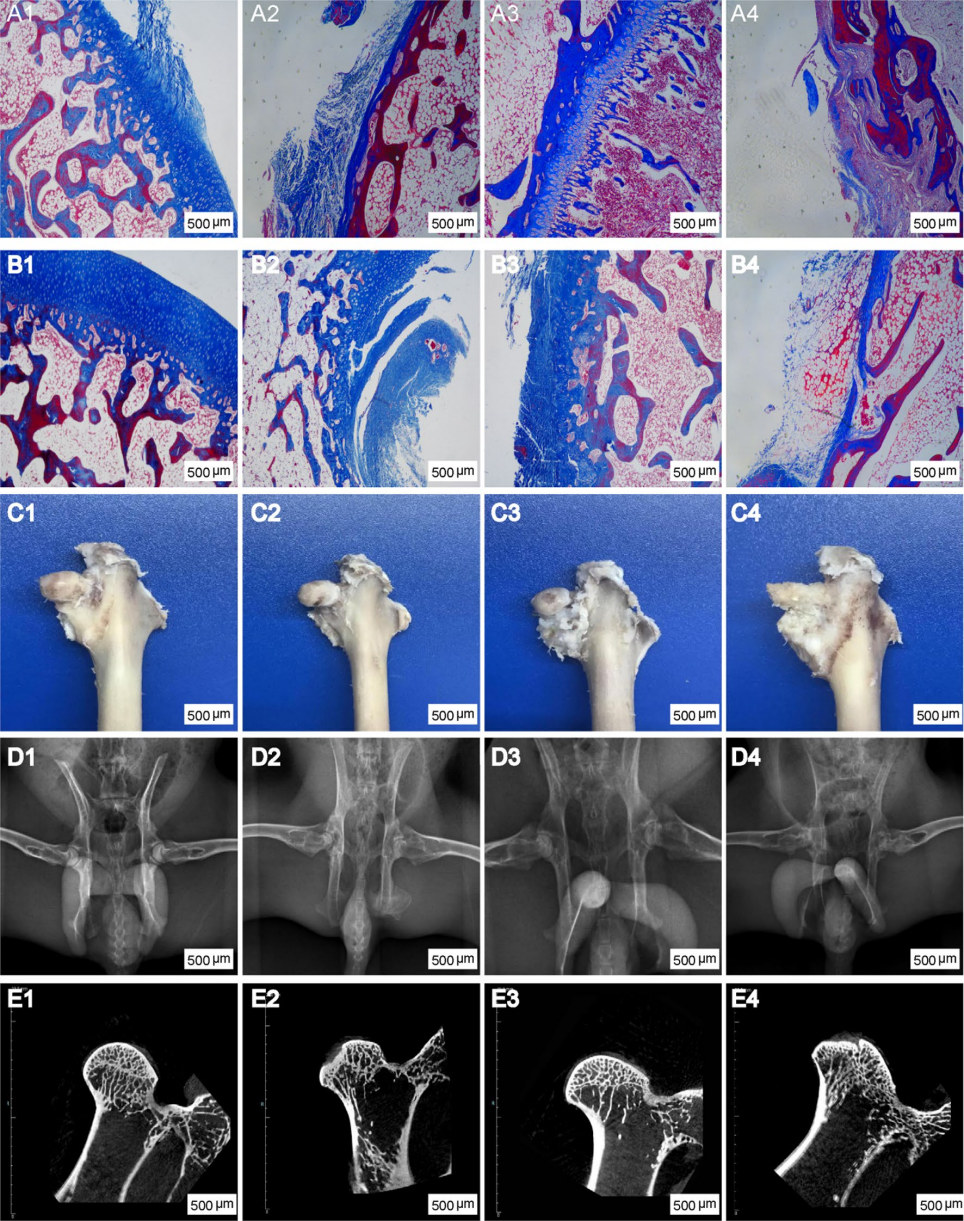
Overexpression of Runx2 promotes osteonecrosis repair. (A1–A4) and (B1–B4) Masson trichrome staining was used to evaluate the repair performance of necrotic femoral head at 3 and 6 weeks respectively; (C1–C4), (D1–D4) and (E1–E4) Gross morphology, X-ray and Micro-CT were used to evaluate the repair performance of necrotic femoral head at 6 weeks; From left to right: Ad-Runx2 group, Runx2-siRNA group, MSCs group and Model group

Masson trichrome staining was used to analysed histological changes of necrotic femoral head at 3 weeks and 6 weeks. The Model group resulted in obvious hollowing and inflammatory tissue at 3 weeks (Fig. 5A4). The osteonecrosis area decreased after 6 weeks, and was covered by few fibrous tissue (Fig. 5B4). Compared with the Model group, the Runx2-siRNA group obtained small level repairment and was covered by few few fibrous tissue and cartilage (Fig. 5A2 and B2). The Ad-Runx2 and MSCs groups obtained better repairment. Large amounts of fibrous tissue and cartilage covered the necrotic femoral head (Fig. 5A1and A3). At 6 weeks, the regenerated tissue of Ad-Runx2 that contained rich cartilage and bone tissue basically completely covered the necrotic femoral head as indicated by Masson trichrome staining (Fig. 5B1).

The gross morphology, X-ray and CT images were used to evaluate the repair performance of necrotic femoral head at 6 weeks. The Model group had obvious bone defect and the morphology can barely be observed(Fig. 5C4). The Runx2-siRNA group showed collapsed femoral head, which has large bone defect and was covered by few cartilage (Fig. 5C2). More regular femoral head that was covered by cartilage appeared in the MSCs group (Fig. 5C3). In the Ad-Runx2 group, the necrotic femoral head was completely repaired and covered by hyaline cartilage, and the regenerated femoral head was smooth and regular (Fig. 5C1). Therefore, the results of gross morphology observation and Masson Trichrome Staining suggested that both MSCs group and Ad-Runx2 group have a obvious repairment, but Ad-Runx2 group produced better outcomes.

In the X-ray and CT images, the Model group showed the destruction of the femoral head, uneven density and disappeared joint space, the trabeculae in the subchondral bone was missing and disordered (Fig. 5D4 and E4); In the Runx2-siRNA group, the femoral head had irregular hemispherical shape, with uneven density and vesicular image, and the joint space was fuzzy, and the trabeculae was non-regular arrangement with massive fracture (Fig. 5D2 and E2); In the MSCs group, the femoral head was basically normal, with a small amount of high density shadow, and the joint space was slightly reduced, and the trabeculae was slightly disordered with a small amount of fracture (Fig. 5D3 and E3); The Ad-Runx2 group had normal morphology, uniform density and clear joint space, and the trabeculae was regular arrangement without fracture (Fig. 5D1 and E1); These results were basically consistent with the molecular experiments in vivo and in vitro.

## Discussion

Bone is a highly vascularized tissue, its blood supply accounts for about 10% of cardiac output. The oxygen concentration of the bone marrow is about 1 ~ 7% [27]. As we all know, the hypoxic microenvironment is often caused by injury or necrosis of bone and its surrounding soft tissue [28, 29]. There is different sensitiveness in the hypoxia microenvironment among osteocytes, osteoclasts and osteoblasts, which means different survival times from 12 to 48 h. The accumulation of metabolites in the osteonecrosis area can aggravate the occurrence of apoptosis and death, and limit the self-repair ability of necrotic bone [30, 31]. As an important source of cell transplantation, MSCs can maintain activity and osteogenic differentiation ability in the hypoxic environment [32–34]. When MSCs are cultured under hypoxia, its proliferative capacity, alkaline phosphatase (ALP) activity, calcified nodule formation, neovascularization, vascularized bone regeneration and production of type I and type III collagen can be significantly induced [35–38]. The evidence provides theoretical support for the survival and differentiation ability of transplanted MSCs.

Osteogenic differentiation is the premise for treating ONFH. To date, many studies have shown that various transcription factors can affect the differentiation of MSCs toward osteoblasts, such as Runx2, osterix, and β-catenin. The Runx2 transcription factor has been proved that it is an essential inducer for bone formation and the osteogenic differentiation of MSCs [39–43]. Gao and his colleagues have demonstrated that Homeobox protein Hox-B7 (HOXB7) can enhance the osteogenic differentiation of MSCs by up-regulating Runx2 [44]. On the contrary, lack or mutation of Runx2 can lead to diseases. Cleidocranial dysplasia (CCD) will happen after mutations of Runx2, and bone formation will be severely impaired after lacking or inhibiting of Runx2 [45–48]. This research has proved that MSCs overexpressing Runx2 can improve osteogenic genes expression and maintain osteoblastic phenotype, and inhibition of Runx2 would lead to converse results.

Recently, some researchers have promoted Runx2 expression to improve bone formation in vivo. Zhao and his colleagues transfected MSCs with Runx2 by adenoviral vectors and implanted MSCs into mice. The data showed MSCs overexpressing Runx2 formed substantially more bone than cells transfected with control virus [49]. Byers and coworkers proposed a new method to improve bone formation through making tissue-engineered constructsusing that consists of three dimensional polymeric scaffolds and MSCs overexpressing Runx2. Constructs were subsequently implanted into calvaria defects in rats, and micro-CT and histomorphometry were used to analyze bone healing. The data showed that Runx2-modified constructs contained twice as much bone as control constructs [50, 51]. In this study, we injected simple MSCs or MSCs that had been transfected with Ad-Runx2 or Runx2-siRNA into ONFH rabbit models, the results showed unsatisfactory results in the Runx2-siRNA group, the femoral head had irregular hemispherical shape, with uneven density and vesicular image, and the joint space was fuzzy, and the trabeculae was non-regular arrangement with massive fracture. The MSCs group achieved a certain repair effect, the femoral head was basically normal, with a small amount of high density shadow, and the joint space was slightly reduced, and the trabeculae was slightly disordered with a small amount of fracture. The Ad-Runx2 group had a better effect, the femoral head had normal morphology, uniform density and clear joint space, and the trabeculae was regular arrangement without fracture, which meant necrotic femoral head was basically completely repaired after 6 weeks. This is the first study to induce MSCs directly by overexpressing Runx2 to treat ONFH.

## Conclusions

Our study showed that overexpression of Runx2 can improve the osteogenic differentiation potential of MSCs, and the repair effect of MSCs for treating ONFH. However, this study has some limitations. Firstly, Some experiments to verify changes of MSCs overexpressing Runx2 have not been completed, such as proliferation and migration. Secondly, more rabbits and detection time may be needed to provide more obvious comparisons.
